# Function of Cancer Associated Genes Revealed by Modern Univariate and Multivariate Association Tests

**DOI:** 10.1371/journal.pone.0126544

**Published:** 2015-05-12

**Authors:** Malka Gorfine, Boaz Goldstein, Alla Fishman, Ruth Heller, Yair Heller, Ayelet T. Lamm

**Affiliations:** 1 Faculty of Biology, Technion- Israel Institute of Technology, Technion City, Haifa 3200003, Israel; 2 Faculty of Industrial Engineering and Management, Technion- Israel Institute of Technology, Technion City, Haifa 3200003, Israel; 3 Department of Statistics and Operations Research, Tel Aviv University, Ramat Aviv, Tel Aviv 6997801, Israel; 4 Tel Aviv, Israel; The University of Chicago, UNITED STATES

## Abstract

Copy number variation (CNV) plays a role in pathogenesis of many human diseases, especially cancer. Several whole genome CNV association studies have been performed for the purpose of identifying cancer associated CNVs. Here we undertook a novel approach to whole genome CNV analysis, with the goal being identification of associations between CNV of different genes (CNV-CNV) across 60 human cancer cell lines. We hypothesize that these associations point to the roles of the associated genes in cancer, and can be indicators of their position in gene networks of cancer-driving processes. Recent studies show that gene associations are often non-linear and non-monotone. In order to obtain a more complete picture of all CNV associations, we performed omnibus univariate analysis by utilizing dCov, MIC, and HHG association tests, which are capable of detecting any type of association, including non-monotone relationships. For comparison we used Spearman and Pearson association tests, which detect only linear or monotone relationships. Application of dCov, MIC and HHG tests resulted in identification of twice as many associations compared to those found by Spearman and Pearson alone. Interestingly, most of the new associations were detected by the HHG test. Next, we utilized dCov's and HHG's ability to perform multivariate analysis. We tested for association between genes of unknown function and known cancer-related pathways. Our results indicate that multivariate analysis is much more effective than univariate analysis for the purpose of ascribing biological roles to genes of unknown function. We conclude that a combination of multivariate and univariate omnibus association tests can reveal significant information about gene networks of disease-driving processes. These methods can be applied to any large gene or pathway dataset, allowing more comprehensive analysis of biological processes.

## Introduction

Copy number variations (CNV) are a part of normal Human genetic variability. Tens of thousands of CNVs have been reported in the Database of Genomic Variants (DGV) based on healthy control samples [1,2]. However, CNVs are also a significant component of variation in disease risk and occurrence of many diseases and disorders, including cancer, HIV infection, autism, and psychiatric diseases [3–5]. In cancer, CNV is one of the most important somatic aberrations found [6]. Nowadays CNV analysis has become a central part of cancer research and many studies concentrate on detecting CNVs in the human genome in normal and diseased tissues and cells. ([7,8], DGV (http://projects.tcag.ca/variation)). In clinics a growing number of CNV are used for diagnostics and personalized therapy.

While individual CNVs can be detected by fluorescent in situ hybridization (FISH), whole genome CNV detection requires microarray-based comparative genomic hybridization (array CGH) or next generation sequencing (NGS) platforms [6]. These platforms generate very high volumes of data, making the analysis very challenging. One major task of CNV data analysis is identifying and characterizing associations between CNVs and diseases, which may potentially be driven by biologically relevant mechanisms [9–11].

Several association studies have been performed for the purpose of linking CNVs to diseases [7,8,12]. For example, Stamoulis et al. [11] focused on monotone relationships between CNV within and across chromosomes; Bussey et al. [12] looked at Pearson’s correlation between CNV and gene expression levels. While most studies associated CNV with gene expression profile, very few, if any, attempts have been made to associate between CNVs of different genes detected in diseased tissue, even though the identification of associations between genes is extremely important for understanding basic biological processes and modeling gene regulatory networks. In this work we undertook such an approach to analyze cancer related CNV data. The rationale was that since CNV formation is part of carcinogenesis, associations between CNVs of genes would be indicative of their roles in carcinogenesis. Additionally, identification of these associations might enable building a gene network of disease driving processes.

To date, the most commonly used association tests are based on Pearson’s or Spearman’s correlation coefficient. Pearson’s test is sensitive to the linear component in a relationship between two variables, while Spearman’s test detects monotone relationships, such as a sigmoid. Hence, both tests are not able to detect non-monotone relationships such as U-shaped, ellipse, sinusoid, etc. Recent studies show that gene associations are often non-linear and non-monotone [13–15]; therefore in order to obtain a complete unbiased picture of all gene associations one must apply other statistical methods.

Recently, several statistical tests for detecting any type of relationships, including non-monotone ones, were proposed. In particular, Szekely et al. [16,17] suggested a test, named dCov, based on distance covariance and distance correlation; Reshef et al. [18] presented a test based on a novel measure of dependence—the maximal information coefficient (MIC); and Heller et al. [19] proposed a test based on ranks of distances, named HHG. Extensive simulation studies comparing between HHG, dCov, MIC, Spearman and Pearson have been performed [13,19]. Their main conclusions were that HHG is typically more powerful than dCov and dCov is usually more powerful than MIC in non-monotone settings.

In addition to their being univariate analysis tools capable of identification of a broad range of association types, dCov and HHG are also applicable for multivariate analysis, i.e., testing for dependence between the variables X and Y, when X and Y are vectors rather than single variables. Thus these tests can be used for identifying associations between pathways or between a gene and a pathway, even when the sample size is much smaller than the dimension of either X or Y.

The second aim of this work was demonstrating the effectiveness of association tests which are also capable of detecting non-monotone relationships, such as dCov, MIC and HHG for analyzing whole genome association data. For this purpose we utilized these tests alongside the standard Spearman and Pearson test in the analysis of CNV data derived from 60 human cancer cell lines (NCI-60) [12]. We have found that the application of tests capable of detecting any type of relationships, such as dCov and HHG, for univariate analysis, results in identification of twice as many associations compared to those found by Spearman and Pearson alone. Most of the new associations were detected by the HHG test. Moreover, multivariate analysis by means of dCov and HHG was able to associate between genes of unknown function from our dataset and basic biological pathways, providing a clue to possible biological functions of these genes.

The methods presented here can be useful in many other settings which require detection of associations of genes and pathways, such as reconstruction of networks and pathways—an important task in systems biology [20]. This study demonstrates that by using these methods researchers can uncover more associations of various types, and thus have a broader picture at their disposal when attempting to study biological phenomena.

## Results

### Identification of Gene-by-Gene Associations

**Fig 1:**
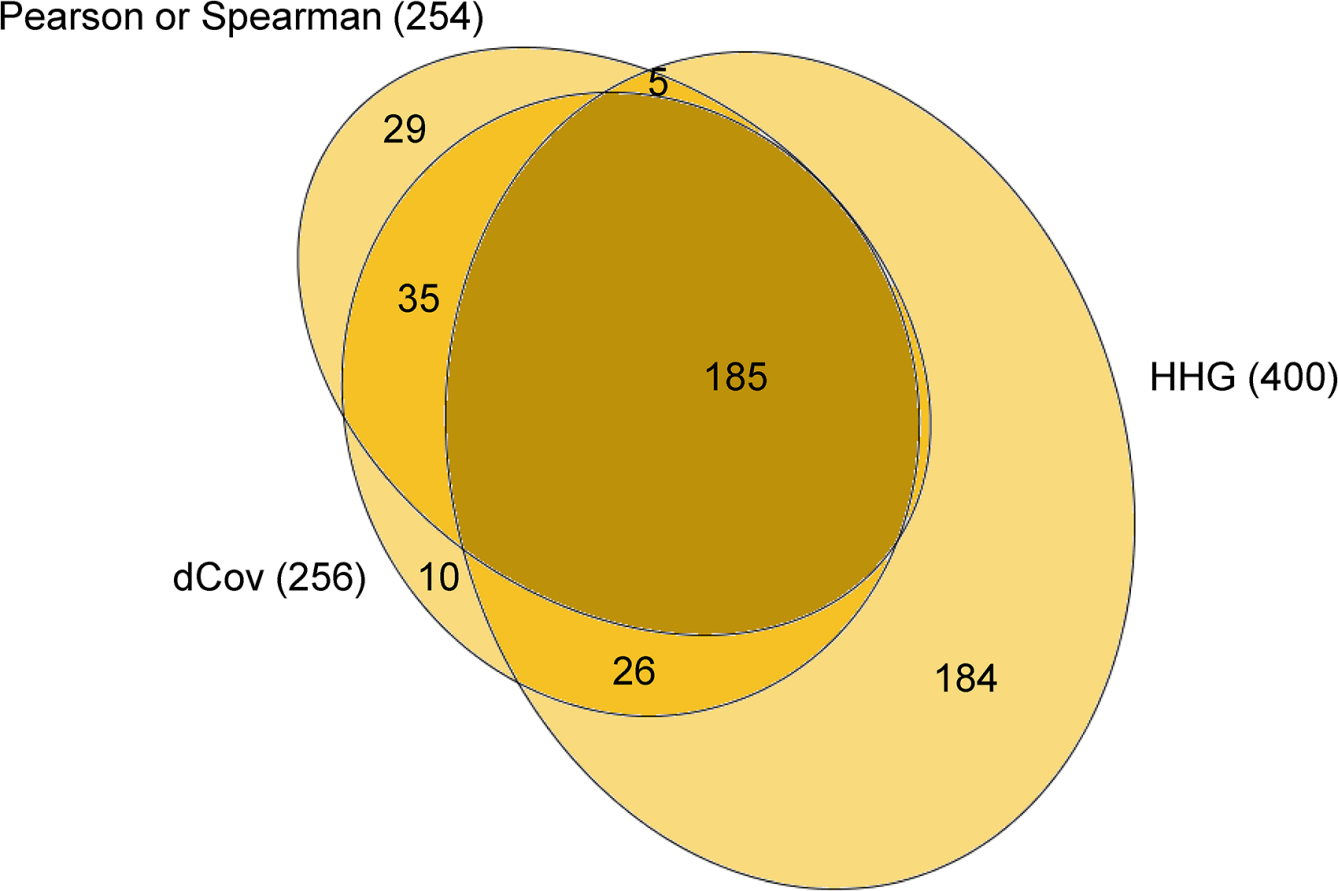
Euler diagram of the significant discoveries found by Pearson or Spearman, dCov and HHG. MIC was excluded due to the small number of significant findings provided by this method. The area of each oval represents the number of significant tests of each method, and intersections (emphasized by different colors) represent common discoveries. Evidently, Pearson or Spearman, dCov and HHG share 185 discoveries; 184 tests were significant by HHG but not by Pearson, Spearman or dCov; 10 tests were significant by dCov and not by Pearson, Spearman or HHG; 29 tests were significant by Pearson or Spearman but not by dCov or HHG; dCov and HHG share 26 discoveries; Pearson or Spearman and dCov share 35 discoveries; and Pearson or Spearman and HHG share only 5 discoveries.

In order to find associations between cancer-related CNVs, we used CNV data obtained by an array CGH from 60 human cancer cell lines (the NCI-60; [12]). Within the CGH array we selected clones that have known gene symbols and, for consistency, no missing values in any cell line. The result contained 99 genes. In addition to the traditional association tests, Spearman and Pearson, we applied three tests, dCov, MIC and HHG, which are also capable of detecting non-monotone relationships. An association was considered significant if the FDR-adjusted p-value was less than 0.05 using the Benjamini-Hochberg procedure [21]. Out of 4851 pair-wise comparisons, Pearson or Spearman detected 254 significant associations, dCov detected 256, MIC detected 157 and HHG detected 400 significant associations (see Fig 1, Table 1, S1 Fig, and S1 Table for detailed results). Comparison of the three tests capable of detecting any type of relationships, namely dCov, MIC and HHG, revealed that they share 139 common significant results. Furthermore, 44 associations were found significant only by dCov; 11 only by MIC and 183 only by HHG (S1 Fig, top-right). Comparing Pearson and Spearman with dCov and HHG revealed that 29 significant associations were discovered solely by Pearson or Spearman, only 10 solely by dCov while 184 were discovered solely by HHG (Fig 1).

**Table 1:** Summary of the significant discoveries (after adjusting for multiple testing) found by Pearson or Spearman, dCov, MIC and HHG.

Pearson or Spearman (254)	dCov (256)	MIC (157)	HHG (400)	Number of discoveries
**V**	**X**	**X**	**X**	29
**X**	**V**	**X**	**X**	9
**X**	**X**	**V**	**X**	10
**X**	**X**	**X**	**V**	**178**
**V**	**V**	**X**	**X**	220
**V**	**X**	**V**	**X**	140
**V**	**X**	**X**	**V**	190
**X**	**V**	**V**	**X**	140
**X**	**V**	**X**	**V**	211
**X**	**X**	**V**	**V**	145
**V**	**V**	**V**	**X**	139
**V**	**V**	**X**	**V**	185
**V**	**X**	**V**	**V**	138
**X**	**V**	**V**	**V**	139
**V**	**V**	**V**	**V**	138

V and X, respectively, indicate whether the method is included or excluded in each comparison. For example, line 1 of the table indicates that 29 tests were found significant only by Pearson or Spearman; line 4 shows that 178 tests were found significant only by HHG; and the last line implies that Pearson or Spearman, dCov, MIC and HHG share 138 common significant findings.

Of the number of significant statistical associations found by dCov, MIC or HHG, but not by Pearson or Spearman, the number found by HHG was exceptionally large. Specifically, while the number of significant associations shared by Pearson or Spearman and HHG is 190, Pearson and Spearman missed 210 associations found by HHG, whereas HHG missed only 64 associations found by Pearson or Spearman. In the above analysis, we combined Pearson's and Spearman's results that had adjusted p-value less than 0.05 as if they were a single method, even though this gives then an advantage compared to other methods. Given this, it is all the more interesting that HHG found 57% more associations then Pearson and Spearman. We therefore conclude that analysis based on the traditional Pearson and Spearman association tests could miss a significant proportion of all possible associations between genes.

**Fig 2:**
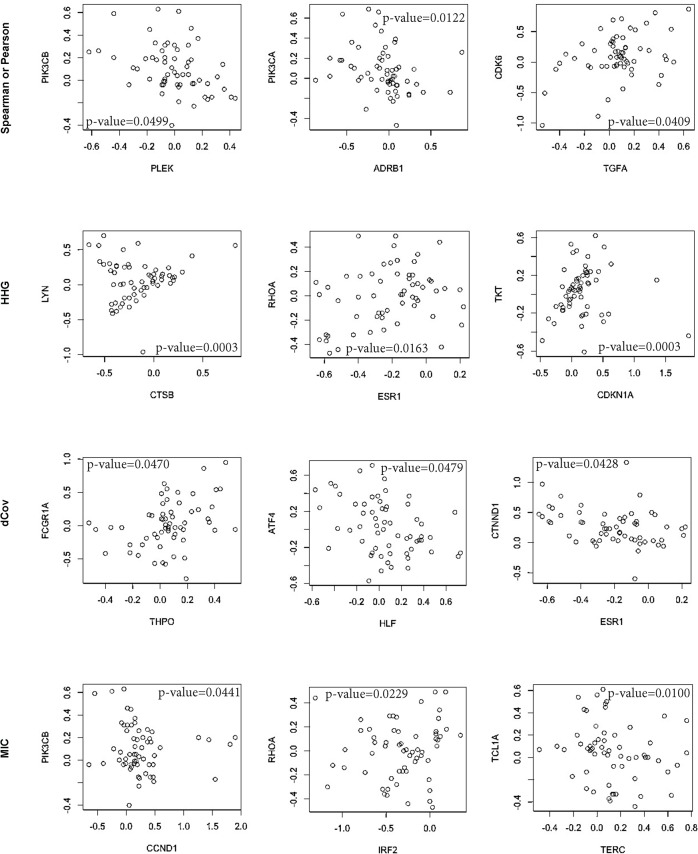
Example of significant relationships. First line consists of three findings discovered only by Spearman or Pearson; second, only by HHG; third, only by dCov; and fourth, only by MIC. P-values (after adjusting for multiple testing) are denoted in each plot.

In order to demonstrate the biological relevance of the associations detected by HHG we took a closer look at the detected associated gene pairs. One example of an association found only by HHG is the association between the genes LYN and CTSB (Fig 2). LYN encodes a non-receptor tyrosine-protein kinase, a regulator of many signal transduction pathways, while CTSB encodes cathepsin B, a thiol protease participating in intracellular degradation and turnover of proteins. No direct biological interactions between these two proteins are known, however they both interact directly with a third protein, Sphingosine kinase 1 (SPHK1). SPHK1 catalyzes the phosphorylation of sphingosine to form sphingosine-1-phosphate (S1P), a key sphingolipid signaling molecule involved in cell growth, survival, differentiation and motility. Interaction between LYN and SPHK1 is essential for the activation of SPHK1 [22]. On the other hand, interaction between Cathespin B and SPHK1 has been shown to down-regulate SPHK1 levels *in vivo* [23] and to cleave it *in vitro* [24]. This experimental data demonstrates that the association between LYN and CTSB identified by HHG is indeed biologically relevant. Moreover, the existence of the association between CNV of LYN and CTSB points to LYN- SPHK1 and CTSB- SPHK1 interactions as being important for carcinogenesis.

Another example for an association found only by HHG is the association between the genes CDKN1A and TKT (Fig 2). CDKN1A codes for CDK-interacting protein 1 (p21), a potent cyclin-dependent kinase inhibitor that regulates cell cycle progression through the G1/S checkpoint. TKT codes for Transketolase, a central enzyme of the Pentose phosphate pathway. The association between CDKN1A and TKT detected by HHG reflects in fact a relationship between the pathways these two genes belong to. Following cell cycle progression from G1 towards the S phase, there is an up-regulation of the Pentose phosphate pathway, which is responsible for production of ribose-5-phosphate (R5P), needed for the synthesis of nucleotides and nucleic acids [25]. All the genes in the examples above are located on different chromosomes or far away from each other on the same chromosome; hence physical proximity cannot explain the CNV-based associations.

### Identification of gene function using multivariate association tests

**Fig 3:**
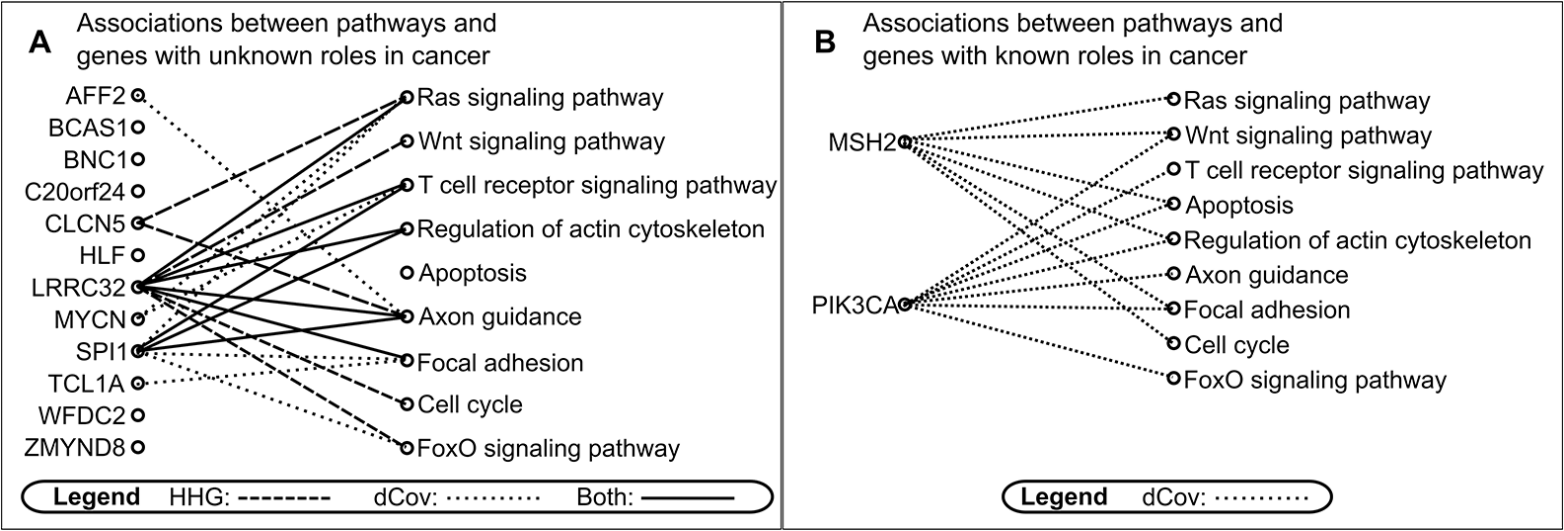
Bipartite graph displaying gene-to-pathway associations, as determined by HHG and dCov. In panels A and B, genes (on the left) and pathways (on the right) were analyzed for association by HHG and dCov. Significant associations (after adjusting for multiple testing) are linked by lines: dashed for HHG, dotted for dCov, and solid for both. A) Significant associations between genes with unknown function and cancer related pathways. Associations found by dCov and HHG are marked. B) Significant associations between genes with known function and cancer related pathways. Only associations found by dCov are shown as no significant associations were found by HHG.

Detection of associations between pairs of genes by univariate analysis is a good start towards deriving biological information from CNV data, as shown above. However, when dealing with a large number of genes, the function and a relation to biological pathways of many genes are often unknown. Finding associations with known genes may shed light on their possible function, but multivariate analysis could provide additional important information. Therefore, we applied the multivariate tests for dependence between several genes of unknown function in our dataset and known pathways, using dCov and HHG multivariate tests. Specifically, of the 99 genes in our dataset, twelve genes have no known function or relation to a biological pathway (Fig 3), as determined by using KEGG pathway ([26,27]; http://www.genome.jp/kegg/tool/map_pathway1.html). To detect their associations with known pathways, we first assigned the rest of the genes to pathways based on KEGG pathway mapper (S2 Table), and then selected eight experimentally proven biological pathways containing at least five genes from our dataset (Fig 3). In addition, the apoptosis pathway, being one of the basic cancer related mechanisms, was included in our study even though only two genes from our dataset have been assigned to it. Next, we tested for associations between each gene-pathway pair among those twelve genes and nine pathways. We applied dCov and HHG which were, of the tests we used above, the only two tests capable of multivariate analysis, i.e., testing for association between vectors (more details are available in the Materials and Methods Section). In total, 108 tests were performed with each method and a test result was considered significant if its FDR-adjusted p-value was less than 0.05 using the Benjamini-Hochberg procedure [21]. Of the twelve genes, six genes showed significant associations to pathways (Fig 3A and S3 Table).

Two genes, LRRC32 and SPI1, were found to be associated with most of the pathways, suggesting they might be signal transduction intermediates, regulating downstream targets belonging to these pathways. These findings are in agreement with the results of the univariate analysis, which significantly associated both genes with serine/threonine kinase PAK1 and SPI1 gene also with HRAS, a GTPase of RAS family. Indeed, according to KEGG pathway mapper PAK1 and HRAS belong to most of the pathways with which LRRC32 and SPI1 were found to be associated. Moreover, both PAK1 and HRAS are involved in transduction of proliferation signals and their miss-regulation leads to abnormal signal transduction and cancer [28,29]. Thus, while a univariate analysis could find association between genes of unknown function and individual genes with known function, the above multivariate analysis could point out their associations with biological processes.

The four remaining associated genes, AFF2, CLCN5, MYCN, and TCL1A, were found to be associated each to one or two specific pathways suggesting they constitute downstream effectors in these pathways (see examples below). No associations were found between the other six genes and any of the pathways.

In the multivariate analysis applied above to genes of unknown function, dCov and HHG discovered similar number of significant multivariate relationships, 15 by dCov, and 13 by HHG, while 8 were detected by both methods. Therefore our analysis did not reveal any clear evidence of superiority of one method over the other in this specific application.

In addition to the multivariate analysis applied to genes of unknown role in cancer, we picked two genes from the dataset, PIK3CA and MSH2, which have established biological function and do not belong to any of the eight pathways according to KEGG, and performed gene-pathway multivariate tests of association by dCov and HHG, similar to those performed above for genes of unknown function. While dCov found 13 significant results, HHG found none (Fig 3B and S4 Table).

**Fig 4:**
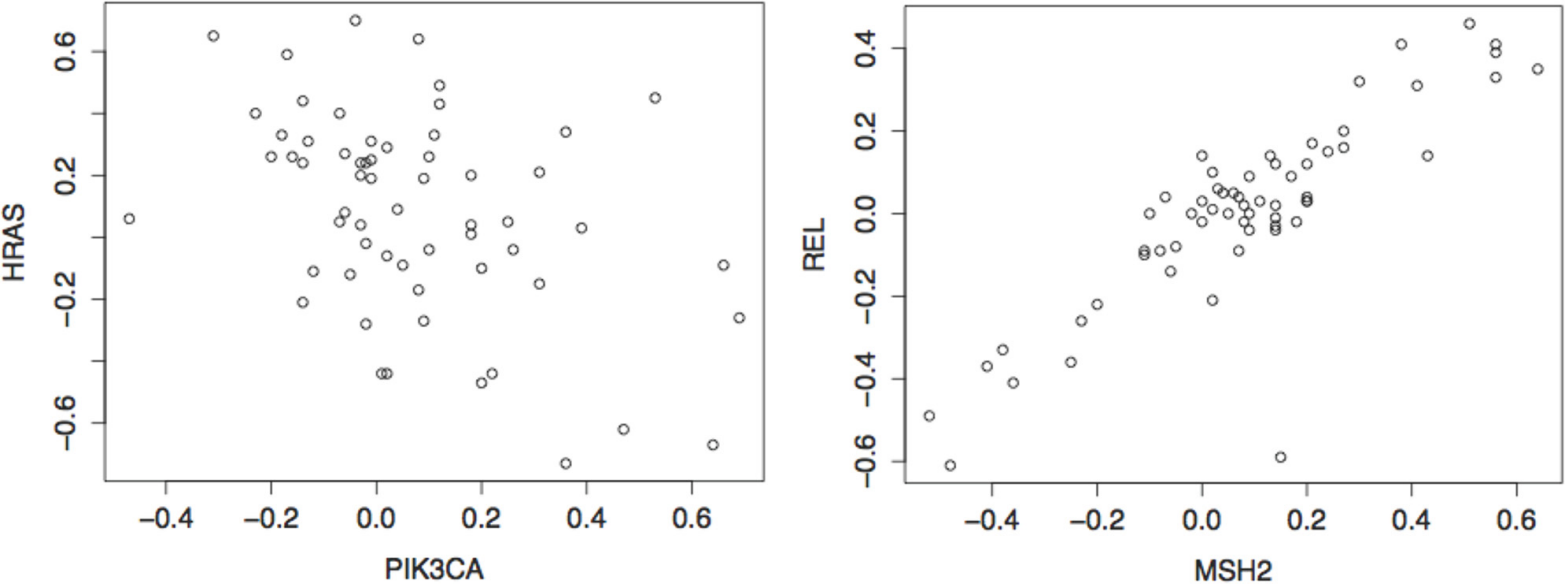
Indication for the linear associations that explains the difference between dCov and HHG in the multivariate analysis with known genes. Scatter plots of PIK3CA versus HRAS (left panel) and MSH2 versus REL (right panel).

The associations, detected by dCov, between MSH2 and cell cycle, apoptosis, focal adhesion, RAS, WNT and actin pathways are consistent with its function in DNA mismatch repair and its connection to cell division [31]. Similarly, associations between PIK3CA, and the following pathways: apoptosis, actin, Focal adhesion, FoxO signaling, T cell receptor signaling, Axon guidance and Wnt (Fig 3B and S4 Table) are supported by vast biological data [32–35]. The relation of PIK3CA to these pathways, as well as its pivotal role in human cancers, is a consequence of it being a key player in activation of signaling cascades involved in cell growth, survival, proliferation, motility and morphology [36]. The discrepancy in the current results of dCov and HHG (Fig 3B) is due to the linear nature of the relationship between these genes and the pathways, and the fact that the strength of HHG is in finding non-monotone relationships. For example, dCov discovered significant association between PIK3CA and the Axon guidance pathway. Looking back at the univariate analysis (S1 Table) we see that PIK3CA was found to be significantly associated with HRAS, which belongs to the Axon guidance pathway, and this association was also found by Pearson or Spearman. Such results indicate strong linear relationship between PIK3CA and HRAS (Fig 4). Similarly, the association found by dCov, but not by HHG, between MSH2 and the Ras signaling pathway can be explained by the significant association found by Pearson or Spearman between MSH2 and gene REL, which belongs to this pathway (S1 Table, and Fig 4). It is expected that known relationships between genes discovered by laboratory methods (such as co-IP) or by bioinformatic analysis of high-throughput data based on classic linear or monotone oriented methods will be strongly biased towards linear or monotone relationships.

Collectively, these results provide a proof of concept for the ability of multivariate analysis to reveal biologically relevant gene-pathway associations.

## Discussion

In this work we undertook a novel approach to whole genome CNV analysis, with the goal being identification of associations between CNV of different genes (CNV-CNV) across 60 human cancer cell lines. We used modern association tests that can detect non-linear and non-monotone associations and applied them in univariate settings, in attempt to identify gene-gene associations. We also used them in multivariate settings, in attempt to identify associations of genes of unknown function with established cancer-related pathways.

Collectively, our univariate analysis demonstrates that associations between CNV of genes found by HHG reflect true biological processes. This suggests that univariate analysis by means of statistical tests which target only linear or monotone associations might result in many biologically important findings remaining unrevealed. Additionally, in this dataset, the superiority of the HHG test over the other tests capable of detecting non-monotone relationships is obvious.

In the multivariate setting, the difference between the highly associated genes (LLRC32 and SPI1) and the other four associated genes is an example of how multivariate analysis can hint at the position of a gene within a pathway. Applied to a larger dataset and combined with univariate analysis, this analysis would allow even more refined positioning of a gene within a pathway.

Six genes did not associate with any of the pathways. This can be due to several reasons; one of them is the limited number of biological pathways with which the genes of unknown function were associated, as a consequence of a limited number of genes (99) with complete CNV data in the database used for this study. Another reason might be the limited biological data reported in KEGG, however this situation is anticipated to improve dramatically in the near future due to continuous accumulation of data from systems biology studies.

In case of LRRC32 and SPI1 discussed above, the univariate and multivariate results complement each other as these genes were found to be associated with pathways by the multivariate analysis and to the specific members of these pathways by the univariate analysis. However it is important to note that this is not a general rule. As a multivariate test of independence identifies dependency between two vectors, while a univariate method only loops over pairs of components and tests for dependency between each pair of variables. Therefore, it is possible to obtain non-significant univariate tests but a significant multivariate test for the same dataset. In fact there is a possibility of no association between any two individual genes and yet of a multivariate association with the full pathway. This can occur due to the combined effects of the variables in the multivariate test. For example, AFF2 was found to be significantly associated with the axon guidance pathway (adjusted p-value = 0.022) by multivariate analysis while no significant associations between AFF2 and any of the genes constituting the axon guidance pathway were found by the univariate analysis. This might be the result of weak associations between AFF2 and pathway members, or alternatively due to a strong association with a pathway member that was not included in the data. In any case, the discovered multivariate analysis gene-pathway association could not have been deduced based on the univariate analysis results.

In the opposite case, two genes, A and B, may be associated by univariate analysis, while no association between gene A and the pathway gene B belongs to is found by multivariate analysis. For example CLCN5 was found by the univariate analysis to be associated with MET and BCL2, both of which belong to the Focal adhesion pathway, which was not associated with CLCN5 by multivariate testing. A multivariate analysis did reveal, however, associations between CLCN5 and the Axon guidance and RAS pathways (Fig 3). Both of these pathways contain MET, the only pathway member found to be associated with CLCN5 by the univariate analysis. Such results are expected since MET is a Receptor Tyrosine Kinase, transducing signals from outside the cell, and thus is at the very start of many pathways, whereas BCL2 is a terminal protein in many pathways. This means that a univariate association with them is not strong enough to detect a pathway association. Corroboration that CLCN5 CNVs are associated with the Axon guidance pathway comes from the observation that 65.9% of central nervous system cancers have a loss of one or two copies of the CLCN5 gene (COSMOS, [30]).

These examples demonstrate the possible advantage of multivariate tests of independence over univariate tests when the goal is finding a relationship between a gene and a group of genes, such as a pathway, or finding an association between two groups of genes (e.g. two pathways). In general, in order to obtain a complete picture, both association tests types should be applied.

The dCov and the HHG tests are permutation tests, and the computation of many such tests can be computationally challenging. Distribution-free univariate tests of a flavor similar to HHG were recently introduced in [37]. These tests can be useful alternatives to the HHG test when a large number of univariate tests are simultaneously examined.

In summary, our results indicate: (1) Multivariate analysis is a very useful tool for ascribing biological roles to genes of unknown function; (2) Univariate omnibus analysis, i.e. using tests that detect all types of relationships, could uncover many new important associations that can not be detected by the common linear and monotone association tests; (3) The HHG test outperformed all the other tests in finding univariate associations; And most importantly, (4) Using a combination of multivariate and univariate associations tests can reveal significant information about gene networks and, in the current context, about cancer-driving processes.

## Materials and Methods

### CNV databases

Comparative genomic hybridization (CGH) data of a panel of 60 human cancer cell lines (the NCI-60) was obtained from [12,38]. The CGH contains 349 clones. After excluding clones with missing values and clones with unknown gene symbols, our analysis was performed on a set of 99 CGH clones, representing 99 genes. S5 Table contains aCGH raw data from NCI-60.

### Univariate analysis

Association analysis was performed on the 99 clones based on their copy number in each of the 60 cell lines from NCI-60. We tested all possible pair-wise associations among the 99 clones, generating 4851 pairs. We used the following tests of independence: (i) test based on Pearson correlation coefficient [39] (ii) test based on Spearman rank correlation coefficient [40] (iii) distance covariance (dCov) [16,17]; (iv) maximal information coefficient (MIC) [18]; and (v) a test based on ranks of distances (HHG) [19]. For each method we adjusted for multiple comparisons by FDR of Benjamini and Hochberg [21], and a test result was considered as significant if its adjusted p-value was less than or equal 0.05.

In the following we provide a summary of the tests. Assume we have *N* independent observations (*X*_*i*_, *Y*_*i*_), *i* = 1, …, *N*, from the joint distribution of (*X*, *Y*), *X*, *Y* ∈ *R* and our goal is to test whether there is a relationship between *X* and *Y*.

#### i. Pearson correlation coefficient

The sample Pearson correlation coefficient, denoted by *r*_*p*_, is given

$$r_{p}=\frac{\sum_{i=1}^{N}\left(X_{i}-\bar{X}\right)\left(Y_{i}-\bar{Y}\right)}{\left(N-1\right)S_{X}S_{Y}}$$

where $S_{X}^{2}=\sum_{i=1}^{N}{\left(X_{i}-\bar{X}\right)}^{2}/\left(N-1\right)$ and $S_{Y}^{2}$ is defined similarly based on *Y*_1_,…,*Y*_*N*_. The value of *r*_*p*_ is between -1 and 1. *r*_*p*_ equals 1 or -1 corresponds to data points lying exactly on a line. A value of 0 implies that there is no linear correlation between *X* and *Y*. If (*X*, *Y*) follows the bivariate normal distribution, under the null hypothesis of no linear relationship between *X* and *Y* (i.e. the true correlation coefficient equals 0), $r_{p}\sqrt{\left(N-2\right)/\left(1-r_{p}^{2}\right)}$ follows a Student’s *t* distribution with *N* − 2 degrees of freedom [39]. This Student’s *t* distribution also holds approximately, if the distribution of (*X*, *Y*) is not normal but the sample size is large enough. We applied this test by using the function cor.test with parameter method = ‘pearson’ in the package *stats* of R (http://www.r-project.org).

#### ii. Spearman correlation coefficient

Spearman correlation coefficient, denoted by *r*_*s*_, is defined similarly to *r*_*p*_ but instead of using the observed values their ranks are used [40]. In case of tied values, a rank equal to the average of their positions in the ascending order of the values is assigned. A value of 1 or -1 for *r*_*s*_ corresponds to the case in which *X* and *Y* are perfect monotone functions of each other. Under the null hypothesis of no monotone relationship between the variables and large sample size, $r_{s}\sqrt{\left(N-2\right)/\left(1-r_{s}^{2}\right)}$ follows a Student’s *t* distribution with *N* − 2 degrees of freedom [40]. We applied this test by using the function cor.test with parameter method = ‘spearman’ in the package *stats* of R (http://www.r-project.org).

#### iii. The dCov test

The distance covariance test [16,17] uses all pairwise Euclidean distances *a*_*ij*_ = |*X*_*i*_ − *X*_*j*_| and *b*_*ij*_ = |*Y*_*i*_ – *Y*_*j*_|, *i*, *j* = 1,…,*N*. Then, the resulting two distance matrices are centered by

$$A_{ij}=a_{ij}-\frac{1}{N}\sum_{i=1}^{N}a_{ij}-\frac{1}{N}\sum_{j=1}^{N}a_{ij}+\frac{1}{N^{2}}\sum_{i=1}^{N}\sum_{j=1}^{N}a_{ij}$$

and

$$B_{ij}=b_{ij}-\frac{1}{N}\sum_{i=1}^{N}b_{ij}-\frac{1}{N}\sum_{j=1}^{N}b_{ij}+\frac{1}{N^{2}}\sum_{i=1}^{N}\sum_{j=1}^{N}b_{ij}.$$

The sample distance covariance, is defined as the average of the componentwise product matrix of the two centered distance matrices: $\sum_{i=1}^{N}\sum_{j=1}^{N}A_{ij}B_{ij}/N^{2}$, and is the test statistic for testing the null hypothesis of independence between *X* and *Y*. The value of the population distance covariance of *X* and *Y* equals zero if and only if they are independent. The dCov test is implemented in the R package *energy* as a permutation test (http://www.r-project.org).

#### iv. The MIC test

The test of MIC [18] is based on the discrete version of the mutual information

$$\sum_{x}\sum_{y}\text{Pr}\left(X=x,Y=y\right)\text{log}\left\{\frac{\text{Pr}\left(X=x,Y=y\right)}{\text{Pr}\left(X=x\right)\text{Pr}\left(Y=y\right)}\right\}.$$

If a relationship exists between two variables, then a grid can be drawn on the scatter plot of the two variables that partitions the data to encapsulate that relationship. Specifically, consider all grids *G* partitioning the *X*-values and *Y*-values into *x* and *y* bins, respectively. Let *I* (*G*; *x*, *y*) be the empirical mutual information of a grid *G* with *x* and *y* bins, such that the probability distribution functions are replaced by the fraction of observations falling in that cell.

Their aim was to use as test statistic

$$M=\underset{\left(x,y\right)}{\text{max}}\left\{\frac{{\text{max}}_{G}\;I\left(G;x,y\right)}{\text{log}\;\text{min}\left\{x,y\right\}}\right\}.$$

In practice, the MIC test statistic is based on a dynamic programming algorithm that only approximates max_*G*_ *I* (*G*; *x*, *y*)/log min {*x*, *y*}, and the outer maximization step in *M* is over (*x*, *y*) such that *xy* < *N* ^0.6^. The MIC test statistic, under the null hypothesis of independence, depends only on the ranks of the data, therefore look-up tables of the quantiles of the null distribution were generated for various sample sizes. The code for applying the MIC test and the look-up tables are available at the MINE website: exploredata.net.

#### v. The HHG test

The rationale of the HHG [19] test is the observation that if *X* and *Y* are associated, closeness in the *X*-values tends to give rise to closeness in *Y*-values. The test is based on the pairwise distances between the sample values of *X* and *Y* respectively, {*d*_*X*_ (*X*_*i*_, *X*_*j*_): *i*, *j* ∈{1,…, *N*}}, {*d*_*Y*_ (*Y*_*i*_, *Y*_*j*_): *i*, *j* ∈{1,…, *N*}}. The only restriction on the distance metrics *d*_*X*_(⋅,⋅) and *d*_*Y*_(⋅,⋅) is that they are determined by norms. For simplicity of notation we consider here identical norm distances for *X* and *Y*, denoted by *d*(⋅,⋅). Consider two fixed observations *i* and *j*, and for *m* = 1,…,*N* let *δ*_*m*_ (*X*_*i*_, *X*_*j*_) = *I*{*d*(*X*_*i*_, *X*_*m*_) ≤ *d* (*X*_*i*_, *X*_*j*_)} and *μ*_*m*_ (*Y*_*i*_, *Y*_*j*_) = *I*{*d*(*Y*_*i*_, *Y*_*m*_) ≤ *d* (*Y*_*i*_, *Y*_*j*_)}, where *I*{*A*} is an indicator function that equals 1 when A is true, and 0 otherwise. For each fixed *i* and *j*, a 2 × 2 contingency table is constructed based on *δ*_*m*_ and *μ*_*m*_, *m* = 1,…, *N*, *m* ≠ *i*, *j*, with entries *A*_11_ (*i*, *j*), *A*_12_ (*i*, *j*), *A*_21_ (*i*, *j*), *A*_22_ (*i*, *j*), where $A_{11}\left(i,j\right)=\sum_{m=1,m\neq i,j}^{N}\delta_{m}\;\left(X_{i},X_{j}\right)\mu_{m}\;\left(Y_{i},Y_{j}\right),$ *A*_12_, *A*_21_, *A*_22_ are similarly defined, *A*_.*k*_ = *A*_1*k*_ + *A*_2*k*_ and *A*_*k*._ = *A*_*k*1_ + *A*_*k*2_, *k* = 1,2. Then, the HHG test statistic is defined as $T=\sum_{i=1}^{N}\sum_{j=1,j\neq i}^{N}S\left(i,j\right)$ where *S*(*i*, *j*) is either Pearson’s chi squared test statistic based on the contingency table given *i* and *j*, namely,

$$S_{p}\left(i,j\right)=\frac{\left(N-2\right)\left\{A_{12}\left(i,j\right)\;A_{21}\left(i,j\right)-A_{11}\left(i,j\right)\;A_{22}\left(i,j\right)\right\}}{A_{1.}\left(i,j\right)A_{.1}\left(i,j\right)A_{2.}\;\left(i,j\right)A_{.2}\left(i,j\right)},$$

or the log-likelihood ratio statistic,

$$S_{LR}\left(i,j\right)=2\sum_{k=1,2}\sum_{l=1,2}A_{kl}\left(i,j\right)\text{log}\left\{\frac{\left(N-2\right)A_{kl}\left(i,j\right)}{A_{k.}\left(i,j\right)A_{.l}\left(i,j\right)}\right\}.$$

In case of zero margin in the contingency table, *S*_*P*_ (*i*, *j*) = 0, and for *S*_*LR*_ (*i*, *j*), a term is zero if *A*_*kl*_ (*i*, *j*) = 0. The sampling distribution of the test statistic under the null hypothesis is computed based on values of *T* under random shuffling of the indices of *X*. The *p*-value of this permutation test is computed by ranking the observed test statistic among the shuffled test statistics. The current analysis is based on Euclidean distances and the Pearson’s chi squared test statistic. The HHG test is implemented in the R package *HHG* (http://www.r-project.org).

### Multivariate analysis

We grouped some of the CGH genes into nine different pathways. We used KEGG Mapper—Search Pathway [26,27] to map genes into pathways and chose only pathways that are not specific to cancer. In addition, we selected pathways with at least five genes. We included the apoptosis pathway even though it has only two genes because of the importance of this pathway in cancer. A separate similar analysis was conducted with MSH2 and PIK3CA genes, which have known function and established biological role in cancer. The aim of the multivariate analysis was to test whether there is an association between any of the genes and the pathways. For the multivariate analysis, we used the dCov and HHG tests, as they are multivariate consistent tests against all alternatives. Pearson, Spearman and MIC are not applicable in multivariate settings. Under the multivariate setting, we let *X* and *Y* be random vectors of lengths *p* and *q*, respectively, and *F*_*X*_, *F*_*Y*_, *F*_*XY*_ denote the respective multivariate cumulative distribution functions of *X*, *Y* and (*X*,*Y*). Under the null hypothesis the vectors *X* and *Y* are independent, namely, *H*_0_: *F*_*XY*_ ≠ *F*_*X*_ *F*_*Y*_, and under the alternative the vectors are dependent, *H*_1_: *F*_*XY*_ ≠ *F*_*X*_ *F*_*Y*_. The dCov and HHG tests were applied using the Euclidean norm as described above. It should be noted that dCov and HHG are applicable under any dimensions *p* and *q*, even for *p* or *q* that are greater than the sample size *N*.

## Supporting Information

S1 Fig Summary of data analysis by area-proportional Euler diagram. The area of each oval represents the number of significant tests found by each method, and intersections (emphasized by different colors) represent common discoveries. The numbers represent the number of significant tests at 0.05 significance level after FDR multiplicity correction.
(PDF)

S1 Table Adjusted p-values calculated by multiple statistical methods. All the genes in our dataset were tested against each other for association. Each pair of genes was tested with Pearson, Spearman, MIC, dCov and HHG, yielding five p-values. The statistically significant (adjusted p-value<0.05) results are marked in yellow.
(XLSX)

S2 Table The pathways used for the multivariate analysis, along with the genes they consist of. A list of pathways obtained from KEGG. Alongside each pathway are the genes from our dataset included within the KEGG pathway. The pathways chosen were those containing at least five genes from our list, or apoptosis—which we considered exceptionally interesting.
(XLSX)

S3 Table Adjusted p-values for testing for association between unknown genes and pathways by dCov and HHG. The pathways in the table were curated from KEGG. A row in the table represents an association test between a pathway and a gene. Each pathway and gene pair appears together with the dCov and HHG adjusted p-values that result from the test. Statistically significant p-values are marked in yellow for HHG and green for dCov. For each gene we tested for association between the gene and the genes in the pathway, and placed the ones yielding statistically significant result (adjusted p-value<0.05) in either HHG or dCov.
(XLSX)

S4 Table Adjusted p-values for testing for association between known genes and pathways by dCov and HHG. The pathways in the table were curated from KEGG. A row in the table represents an association test between a pathway and a gene. Each pathway and gene pair appears together with the dCov and HHG adjusted p-values that result from the test. Statistically significant p-values are marked in yellow for HHG and green for dCov. For each gene we tested for association between the gene and the genes in the pathway, and placed the ones yielding statistically significant result (adjusted p-value<0.05) in either HHG of dCov.
(XLSX)

S5 Table aCGH Raw data from NCI-60 that was used in this paper. (TXT)
