# Supplementary figures and images for: Function of Cancer Associated Genes Revealed by Modern Univariate and Multivariate Association Tests
Source: PLoS One. 2015 May 12;10(5):e0126544. doi: 10.1371/journal.pone.0126544 (PMC4429101; doi:10.1371/journal.pone.0126544)

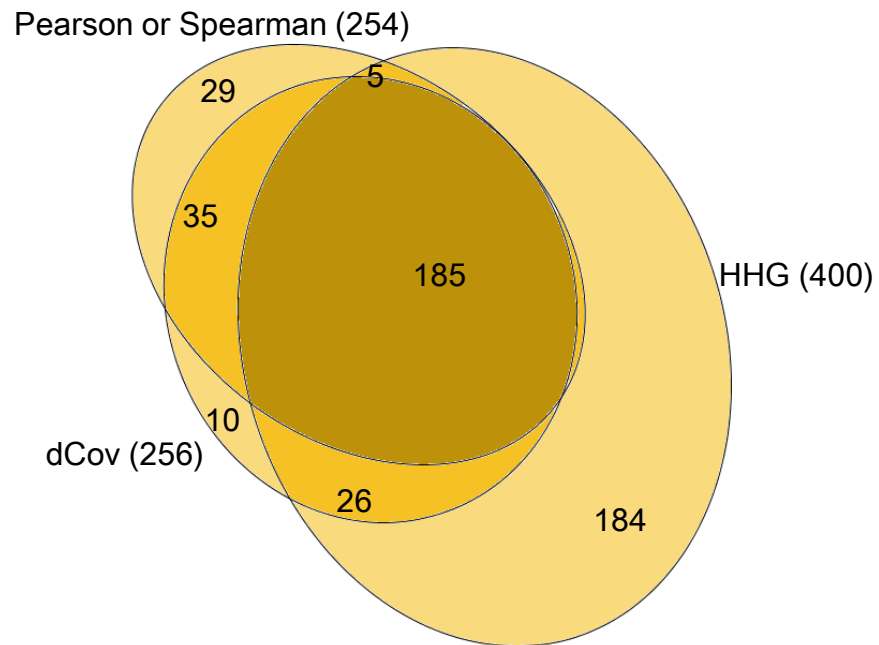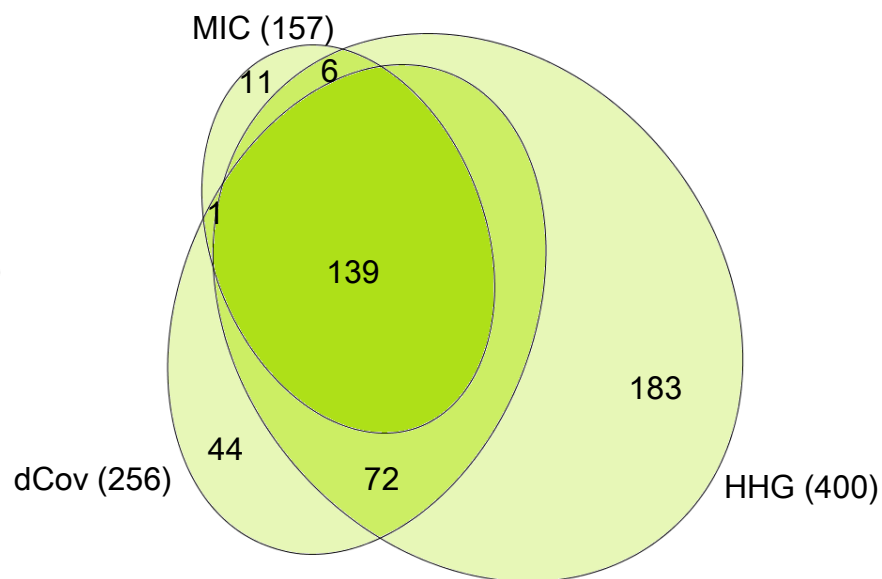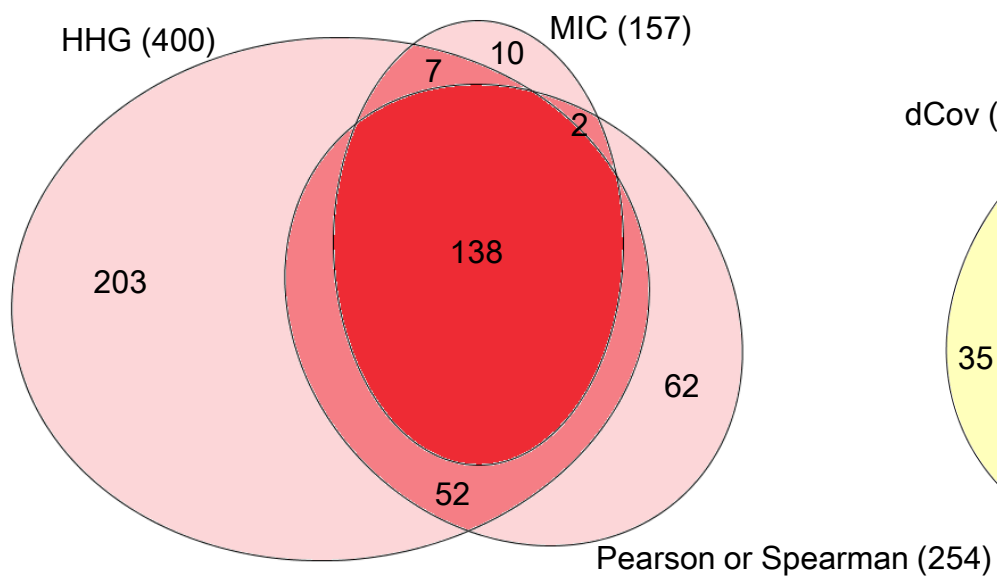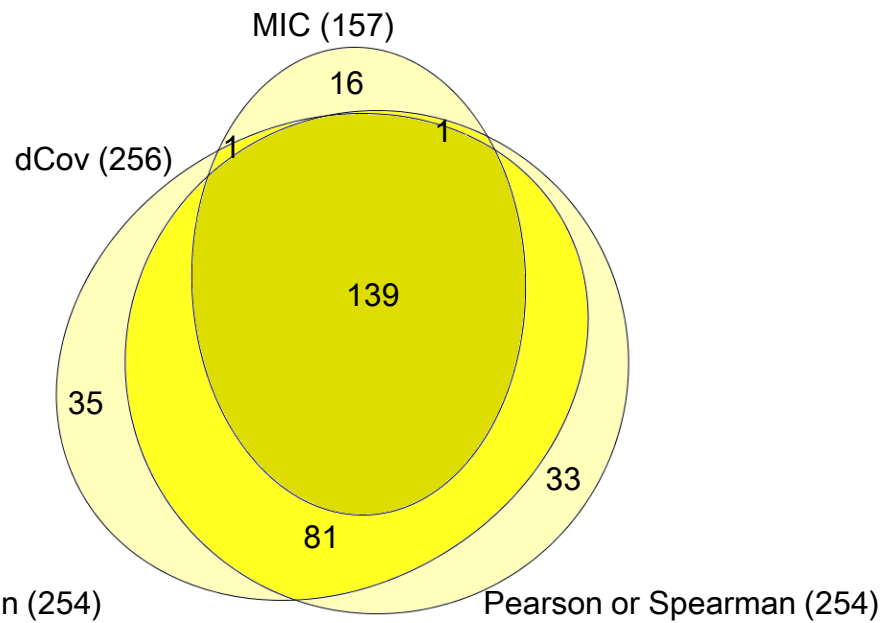

Supplement: S1 Fig — The area of each oval represents the number of significant tests found by each method, and intersections (emphasized by different colors) represent common discoveries. The numbers represent the number of significant tests at 0.05 significance level after FDR multiplicity correction. (PDF) [file pone.0126544.s001.pdf]
